# Mannan Oligosaccharides in Nursery Pig Nutrition and Their Potential Mode of Action

**DOI:** 10.3390/ani2020261

**Published:** 2012-05-23

**Authors:** Veronika Halas, Imre Nochta

**Affiliations:** 1 Department of Animal Nutrition, Faculty of Animal Science, Kaposvár University, P.O. Box 16, H-7400 Kaposvár, Hungary; 2 Provimi, P.O. Box 1, H-8112 Zichyújfalu, Hungary; E-Mail: inochta@hu.provimi.com

**Keywords:** nursery pigs, mannan oligosaccharides, gut integrity, immune response, growth performance

## Abstract

**Simple Summary:**

The aim of the paper is to provide a review of mannan oligosaccharide products in relation to their growth promoting effect and mode of action. Mannan oligosaccharide products maintain intestinal integrity and the digestive and absorptive function of the gut in the post-weaning period in pigs and enhance disease resistance by promoting antigen presentation. We find that dietary supplementation has growth promoting effects in pigs kept in a poor hygienic environment, while the positive effect of MOS is not observed in healthy pig herds with high hygienic standards.

**Abstract:**

Mannan oligosaccharides (MOSs) are often referred to as one of the potential alternatives for antimicrobial growth promoters. The aim of the paper is to provide a review of mannan oligosaccharide products in relation to their growth promoting effect and mode of action based on the latest publications. We discuss the dietary impact of MOSs on (1) microbial changes, (2) morphological changes of gut tissue and digestibility of nutrients, and (3) immune response of pigs after weaning. Dietary MOSs maintain the intestinal integrity and the digestive and absorptive function of the gut in the post-weaning period. Recent results suggest that MOS enhances the disease resistance in swine by promoting antigen presentation facilitating thereby the shift from an innate to an adaptive immune response. Accordingly, dietary MOS supplementation has a potential growth promoting effect in pigs kept in a poor hygienic environment, while the positive effect of MOS is not observed in healthy pig herds with high hygienic standards that are able to maintain a high growth rate after weaning.

## 1. Introduction

Mannan oligosaccharides (MOSs) are often referred to as a potential alternative for antibiotic growth promoters. Although they are non-digestible oligosaccharides, the mode of action of MOSs differs from other prebiotics. By definition, prebiotics are non-digestible components of feed that stimulate the growth and/or activity of beneficial bacteria in the digestive system and promote the gut and general health of the host [1]. MOSs have recently been assigned to nutricines based on the reasoning that they are not direct nutrients either for intestinal microbiota or for the host, but potentially have a positive effect on the health and performance of farm animals [2]. 

The first experience with mannan products was investigating their potential to adhere to the mannose specific lectin on the surface of *E. coli* [3]. Results of *in vitro *studies suggested that dietary MOS could reduce the colonization of pathogenic bacteria in the gut, which was indeed confirmed later in animal, particularly poultry trials [4,5]. Dietary MOS supplementation became prevalent in the 90s as a growth promoter in broiler and turkey feeding [6,7] and to a lesser extent in pig feeding [8]. Relevant data show that mannan products can be efficiently used in two critical periods of swine production, *i.e.*, in piglets during the nursery period and in sows during late gestation and lactation. Numerous publications reported that supplementation of the sow diet with dietary MOS (2 g/kg or 5 g/day/sow) in the last 2–3 weeks of gestation and during lactation improved the growth rate of piglets [9,10,11]. The data on weaned pigs are less consistent in this respect. The mode of action of mannan-containing products has been investigated for approximately 20 years and the following underlying mechanisms have been proved: mannans potentially affect (i) the intestinal micro biota, (ii) the morphology of gut tissue and thus the digestibility of nutrients, (iii) the immune response of farm animals; and (iv) the supposed toxin-binding ability of mannan containing yeast cell derivates. This latter, however, is attributed principally to the β-glucan content [12], therefore in the present paper this property is not discussed. Based on the effects listed above, it is suggested that dietary MOS is able to support gut recovery of piglets after weaning. 

Excellent reviews have been published on the dietary effect of MOSs [12,13,14]; however, a few details of the underlying mechanisms remained unexplained. Since some recent results have filled gaps in our knowledge, the aim of the present paper is to provide an overview on MOS products in relation to their growth promoting effect and mode of action based on the latest publications. 

## 2. Post-Weaning Changes within the Gastro Intestinal Tract of Pigs

Weaning is a stress for the piglets that is usually associated with a dramatic feed refusal. The immediate post-weaning anorexia results in the alteration of gut integrity leading to different physiological changes in the gastro intestinal tract (GIT), such as morphological and functional changes, shift in microbiota population, and increased production of inflammatory cytokines (e.g., reviewed by Dong and Pluske [15]). Even a short period of starving or malnutrition (2–3 days) results in villus atrophy [16] that weakens the absorption capacity of the intestine by reducing the surface area of the gut wall. It is also well known that the brush border enzyme (amino-peptidase N, dipeptyl-peptidase-4 [16]; sucrase [17]) and pancreatic enzyme activity (trypsin, chymotrypsin, amylase, lipase [18]) decline drastically right after weaning. The temporal changes induced in the gut after weaning can be divided into an acute phase of 5–7 days and an adaptive phase of 9–10 days [19]. The changes in the acute phase definitely result in a poorer digestibility of nutrients. The higher rate of undigested nutrients, and particularly of protein, may lead to undesirable processes in the hindgut. The fermentation of N-containing compounds in the digesta yields ammonia that increases the gut pH and supports pathogenic bacteria. Since the microbiota in young piglets is unstable, the ability of the gut flora to block the colonization of harmful species is inadequate. Moreover, neither the innate nor the acquired immune defense of a 4-week old pig can adequately respond to a pathogen challenge. Due to the low antibody concentration of sow milk at late lactation, the level of maternal antibodies is also low in the gut and blood of piglets. This is concurrent with insufficient own antibody production at 4 weeks of age, which only begins to increase at 6–7 weeks of age.

These changes often result in post-weaning diarrhea and a drastic depression in growth rate. In the past, antibiotic growth promoters efficiently prevented the complex post-weaning symptoms. Nowadays all feed additives that promote gut health, sustain eubiosis in the intestine or boost the immune defense of the pigs can be effectively used in nursery feeds. MOSs are suggested to be one of the promising alternatives for antibiotic growth promoters.

## 3. Source and Chemical Traits of Mannan Products

MOSs are non-digestible carbohydrates that are composed of mannose blocks and can be found in the yeast cell wall in complex formation. The composition of the yeast cell is determined by the species, the growth phase and the environmental factors of fermentation. The cell wall is approximately 25–30% of the dry weight of the cell. *Saccharomyces cerevisiae* is a well-known yeast in the bakery and brewery industries and its derivate is used exclusively as MOS product in animal nutrition. The cell wall of *S. cerevisiae *contains both mannan-proteins and β-glucans. The main constituents of the outer cell wall are mannan polymers with α(1-6) and α(1-2) bonds or to a lesser extent α(1-3) bounded side chains (see more details in [12]). The enzymes of the host or of the intestinal bacteria are unable to cleave these bonds and thus MOS has no direct nutritive value, but it has been shown to be able to maintain the gut health. It can be concluded from the relevant literature that although mannan-containing feed additives are almost exclusively derivates of *S. cerevisiae*, due to the processing and production technology, their chemical composition and therefore biological efficiency might be different [13].

## 4. Mode of Action of Dietary MOS

### 4.1. The Effect of Dietary MOS on the Intestinal Microbiota

The development of the beneficial microbiota and sustainment of eubiosis play a crucial role in the defense mechanisms and gut health. There is increasing evidence suggesting that, unlike in young pigs, the composition of the GIT microflora in a healthy adult host remains remarkably stable [20]. The steady state can, however, promptly change at immune suppression or during infection. In general, supporting the growth of *Bifidobacteria* and *Lactobacilli* in the hindgut is supposed to have a positive impact on the host; however, in stress situations, such as weaning, the number of beneficial bacteria often declines, while the number of harmful species, like *E. coli* and *Salmonella,* increases. *In vitro* studies show that in the presence of mannan products the enteric pathogens attach to the mannan compounds in the gut lumen instead of the epithelia, which reduces their colonization. Results of relevant studies suggest that dietary MOS supplementation can reduce the number of harmful bacteria in the hind gut if the pathogen exposure was high, such as in post-infection [21,22,23,24]. A small number of other trials, however, failed to prove that the number of (facultative) pathogens was indeed affected by dietary MOS [25,26]. Singboottra [27] concluded that subject to their chemical structure (bonds and proportion of mannose) the efficiency of mannan products might differ in regard to their potential to moderate the number of *E. coli* and *Salmonella typhimurium*.

The microbiota exists in a dynamic state; increasing the number of any bacterial specie may result in the decrease of another specie. Some studies show that the reduction in the number of pathogenic bacteria in response to dietary MOS supplementation was indeed associated with an increase of beneficial flora, particularly lactobacilli [21,23,28], but this finding is not consistent throughout the literature [24,29,30]. Sims *et al.* [5] found in turkey that dietary MOS supported the growth of *Enterococcus*. These bacteria produce not only short chain fatty acids but also bacteriocin and enterocin and thus enhance the competition and development of beneficial flora [31]. Other results also confirm the positive effect of MOS, since it is reported to reduce the ammonia concentration in the gut [32]. 

Literature data show that dietary MOS supplementation can efficiently reduce the number of pathogens post-infection; however, it is unable to modify consistently the quantity of harmful species under adequate hygienic conditions. The shift in population of beneficial bacteria is not consistent in the different studies, therefore it can be concluded that although dietary MOS may support the maintenance of eubiosis, it probably has no real prebiotic effect. 

### 4.2. The Effect of Dietary MOS on Gut Morphology

The first studies proving that MOS supplementation has a significant impact on gut morphology were conducted with broilers [33]. Results showed that dietary mannan products increase the villous height: crypt depth ratio in young broilers [33] and turkey [14] and also in weaned piglets [34,35]. In a recent study with nursery pigs, Poeikhampha and Bunchasak [36] found that 3 g MOS/kg in the diet resulted in increased crypt depth in the jejunum. An increased villous height: crypt depth ratio is generally associated with a bigger absorptive surface; this ratio is, however, usually reduced during the initial post-weaning period [37]. There are several hypotheses on the beneficial effect of MOS on intestinal morphology, but not all of them were proven in swine. As discussed earlier, the reduction in the *Enterobacteria* population [24] and/or increase in beneficial flora [21,23,28] enhance the short chain fatty acid production in the intestine, which positively affects the recovery of the epithelia. A number of studies reported that dietary MOS supplementation increases the lactic acid and/or volatile fatty acid production in the hind gut [5,28,36]. The microflora as a whole has a trophic effect on the epithelium, which can lead to a faster turnover rate [20]. In particular, butyric acid has anti-inflammatory property, alleviating the hypersensitive reaction of the gut wall associated with the post-weaning period. In turkey, the increased production of the mucus gel layer [14], and in pigs, a prompt recovery of the intestinal mucosal cells [38], was promoted by MOS. Moreover, enhanced gut maturation was reported in broilers [33,39].

The post-weaning morphological changes in the gut might be alleviated if the pig feed contains MOSs. It seems, however, that the structural changes of the epithelial cells are associated with functional changes of the gut tissue. Kim *et al.* [40] found slightly better apparent ileal amino acid digestibility for valine, isoleucine, leucine, lysine and arginine when the piglet diet was supplemented with 1 g MOS product/kg of feed, but the differences were statistically not significant. Nochta *et al.* [41] reported that supplementation of a mannan containing feed additive significantly improved the apparent ileal digestibility of nutrients, particularly that of indispensable amino acids (Lys, Met, M+C, Thr), Ca and P (Table 1). When the diet was supplemented with MOS at the rate of 2 g/kg, the apparent ileal digestibility of nutrients was similar to that in the treatment containing an antibiotic growth promoter. However, further increase of MOS (4 g/kg) did not further improve the ileal digestibility data. In addition to a lessened erosion of the absorptive surface, the better digestibility might be explained at least partly by the lower pH attributed to the more active fermentation by hind gut bacteria. Beneficial microflora produces short chain fatty acids that reduce the pH of the ileal digesta, which can thus result in higher protein hydrolysis and improved protein and amino acid, as well as Ca and P digestibility [41]. The relatively big improvement in apparent threonine digestibility generally indicates a decreased endogenous threonine excretion. 

**Table 1:** The effect of dietary mannan oligosaccharide (MOS) supplementation on the apparent ileal digestibility of nutrients in weaned pigs (%) [41].

	T R E A T M E N T S *					
	M0	M1	M2	M4	AB	RMSE
Dry matter	76.4 ^b^	77.3 ^ab^	79.2 ^a^	77.9 ^ab^	77.2 ^ab^	1.9
Crude protein	72.5 ^c^	73.6 ^bc^	77.5 ^a^	76.3 ^ac^	77.5 ^a^	2.0
Crude fat	93.9 ^a^	93.0 ^a^	93.8 ^a^	93.8 ^a^	94.1 ^a^	0.8
N-free extract	79.6	80.7	81.2	80.9	79.8	2.3
Calcium	47.1 ^b^	55.5 ^a^	54.2 ^ab^	53.9 ^ab^	50.2 ^ab^	5.1
Phosphorus	55.1 ^b^	62.8 ^a^	64.7 ^a^	65.2 ^a^	61.0 ^a^	3.2
Lysine	74.1 ^b^	74.5 ^b^	79.3 ^a^	76.1 ^b^	79.5 ^a^	1.7
Methionine	84.4 ^c^	89.5 ^a^	88.0 ^ab^	86.9 ^b^	82.8 ^c^	1.4
Cystine	54.0 ^c^	74.8 ^a^	72.2 ^a^	65.0 ^b^	74.1 ^a^	3.1
Met+Cys	72.7 ^d^	83.8 ^a^	81.9 ^ab^	78.5 ^c^	79.4 ^bc^	2.0
Threonine	59.1 ^c^	70.4 ^ab^	71.4 ^a^	66.6 ^b^	68.2 ^ab^	2.9

^*^ M0: negative control without antibiotic or MOS; M1: supplementation of 1 g AgriMos/kg feed; M2: supplementation of 2 g AgriMos/kg; M4: supplementation of 4 g AgriMos/kg feed; AB: antibiotic growth promoter containing feed with 0.2 g Maxus-200 (40 ppm avilamycin supplementation)/kg feed; RMSE: Root mean square error. ^a,b,c,d^ common letters within rows indicate no differences at *P* < 0.05.

Therefore, the data shown in Table 1 suggest that the endogenous protein loss can be reduced by MOS supplementation likely due to a faster recovery of the intestinal mucosal cells [38]. Due to the high threonine content of gut cell and mucus protein, the ileal threonine excretion can be twice as high as the lysine or methionine+cystine excretion [42]. The higher threonine digestibility might be associated with a lower turnover of the gut wall layer and less endogenous N losses. However, there are no data available in the literature that would report a reduced endogenous protein and/or threonine loss when dietary MOS is fed.

In conclusion, dietary mannan products can improve the gut morphology in the post-weaning period, which in turn has a positive impact on nutrient supply and on the first line of defense in nursery pigs.

### 4.3. The Effect of Dietary MOS on the Immune Response of Weaned Pigs

Dietary MOS has both an indirect and a direct effect on the immune response of farm animals. According to the fact that the microbiota can modulate the local immunity of the host, the MOS induced shift in gut flora may result in the changes of certain immune variables. 

More evidence has been reported that addition of dietary mannan products directly enhance the immune competence of pigs, particularly that of sows and weaned pigs. Numerous studies with rats, dogs and chickens show that dietary MOS enhances the secretory IgA in different segments of the intestinal mucosa [43,44,45]. The higher mucosa IgA production is likely attributable to an activation of the local immune defense through the mannose binding receptors located in the gut surface. Davies *et al.* [46] reported that 21 days of feeding 2 g of phosphorylated mannan per kg diet altered the T lymphocyte repertoire of jejunal lamina propria. The local immune function initiates the systemic immune response of the host, which is frequently reported in response to MOS supplementation.

Recent results of the present authors show that the non-specific cellular immune variables are modulated by dietary MOS, and the level of supplementation is determining in this respect (Table 2). Nochta *et al.* [47] found that a lower dose of MOS (1 g/kg) increased the responsiveness to lymphocyte stimulation test (LST) with non-specific mitogens (pokeweed mitogen: PWM, Concavalin A: ConA and phytohaemagglutinin: PHA) in weaned pigs; bigger doses (2 or 4 g/kg), however, had no influence or even impaired it. Although Davis *et al.* [48,49] did not prove that MOS increases the responsiveness of LST with PWM or PHA mitogens, their results with regard to the ratio of CD4+/CD8+ lymphocytes could also indicate that MOS supplementation enhances the establishment of a mature T cell repertoire within the gastrointestinal tract of 3-weeks old weaned pigs. Moreover, in the study of Davies *et al.* [46] the percentage of neutrophils tended to increase and percentage of lymphocytes significantly increased in the peripheral blood when piglets were fed 2 g of mannan product/kg feed. The authors supposed that the alteration in systemic immune function was possibly an indirect response to changes that were occurring in the gastrointestinal immunity [46]. 

Recent results suggest that it is worth supplementing dietary mannan in case of an immune challenge. Dietary MOS supplementation (1 g MOS/kg feed) enhanced the specific immune response, particularly the virus neutralization, 2 weeks after immunization with inactivated Aujeszky virus in a study carried out with weaned pigs [47]. In agreement with those results, Franklin *et al.* [50] reported that the specific immunity was enhanced by MOS supplementation as evidenced by greater serum rotavirus neutralization titers in cows supplemented with 10 g MOS daily compared with control cows. The humoral immune response is the result of the activation of the B-cells responsible for the production of antigen specific immunoglobulin. According to a serial study, White *et al.* [21] reported that the serum IgG concentration tended to increase in early weaned piglets fed with mannan containing yeast. Moreover, Shashidhara and Devegowda [51] found significantly higher specific antibody titers in the serum of broiler breeders after vaccination with bursal disease virus when the diet was supplemented with 0.5 MOS g/kg feed. 

**Table 2:** Effect of dietary MOS supplementation on non-specific and specific cellular immune response of weaned pigs (lymphocyte stimulation index) [47].

	T R E A T M E N T S *							
Time (Wks)	M0	M1	M2	M4	AB	NI	RMSE	*P*-value **
Con A								
1	5.08	5.10	5.13	5.10	5.08	-	0.054	0.19
2	5.21	5.21	5.20	5.20	5.17	-	0.122	0.96
5	5.21 ^b^	5.37 ^a^	5.27 ^ab^	5.24 ^ab^	5.20 ^b^	-	0.105	0.02
PHA								
1	3.73	3.72	3.76	3.69	3.71	-	0.071	0.45
2	3.81	3.86	3.81	3.78	3.80	-	0.146	0.84
5	3.81 ^ab^	3.98 ^a^	3.86 ^ab^	3.78 ^b^	3.86 ^ab^	-	0.134	0.02
PWM								
1 ^†^	2.89	2.91	2.90	2.90	2.90	-	0.070	0.96
4	3.06	3.17	3.09	3.12	3.07	-	0.125	0.34
5	3.06 ^a^	3.27 ^b^	3.18 ^ab^	3.13 ^ab^	3.10 ^ab^	-	0.148	0.048
Auj								
1	0.88	1.01	1.01	1.00	1.01	1.01	0.135	0.56
2 ^†^	1.28	1.34	1.24	1.28	1.19	1.02	0.274	0.10
3	1.57 ^ab^	1.95 ^a^	1.55 ^ab^	1.56 ^ab^	1.40 ^bc^	1.04 ^c^	0.339	0.0001
4 ^†^	2.62 ^a^	2.82 ^a^	2.61 ^a^	2.50 ^a^	2.53 ^a^	1.03 ^b^	0.736	0.0001
5	2.94 ^a^	3.15 ^a^	3.23 ^a^	3.11 ^a^	2.78 ^a^	1.04 ^b^	0.686	0.0001

RMSE: root mean square error; M0: negative control without antibiotic or MOS; M1: supplementation of 1 g AgriMos/kg feed; M2: supplementation of 2 g AgriMos/kg; M4: supplementation of 4 g AgriMos/kg feed; AB: antibiotic growth promoter containing feed with 0.2 g Maxus-200 (40 ppm avilamycin supplementation)/kg feed; NI: non-immunized group fed no supplementation. ^a,b,c^ common letters within rows indicate no differences at *P* < 0.05. ** There was no effect of interaction between treatment and replication. ^†^ Replication effect was significant (*P* < 0.05).

The earlier and stronger immune response is essential for the livestock in order to moderate or eliminate the antigen attack. There are two potential modes of action of dietary MOS as discussed by Newman [52] and Franklin *et al.* [50]. In the present paper those pathways are summarized briefly. The first underlying mechanism involves the presence of a collectin, a mannose-binding protein in the blood serum that may act as opsonin. Opsonins are molecules that make foreign antigens more susceptible to the action of the phagocytes. Mannose-binding proteins may bind to mannose-containing structures of a number of viruses and bacteria and trigger the complement cascade of the host immune system [52]. Nielsen *et al.* [53] reported increased presence of mannose-binding proteins in chickens during virus infection. It is likely that MOS stimulates the production of mannose-binding proteins resulting in improved phagocytosis, activation of the complement system, and enhancement of the immune response [50]. The other possible mode of action of MOS involves the natural production of antimannan antibodies [50]. The antimannan antibodies are directed against an oligosaccharide-based epitope of the viruses and microbes and these carbohydrate-specific antibodies may be produced during a normal immune response against the intestinal microflora. Dietary MOS probably enhances the production of these antimannan antibodies at the gut level, which in turn may enter the blood stream allowing for an enhanced response to a viral challenge [21,47]. 

In a recent study with 3-week old weaned pigs, Che *et al.* [54] found that MOS supplementation was associated with rapidly increased numbers of leukocytes, lymphocytes, and neutrophils at the early stage of porcine reproductive and respiratory syndrome (PRRS) virus infection (7 days post-infection). Results of the same study and an earlier trial conducted with mice suggest that dietary MOS has the potential to alleviate inflammation and has an anti-allergic effect, caused by the activation of cellular immunity [55]. Ozaki *et al.* [55] reported lower number of peritoneal acidophils in MOS-fed mice compared to that in control diet-fed ones, moreover MOS treatment reduced interleukin-10 production and tended to suppress ovoalbumin-specific IgE in serum. Che *et al.* [56] supported evidence on the potential of dietary MOS (2 g/kg) to alleviate the hypersensitive reaction post-infection. It has been proved that MOS down-regulates the expression of non-immune and immune genes in pig leukocytes, perhaps providing benefits by enhancing the pig’s immune responses to an infection, while preventing over-stimulation of the immune system [56,57]. It also altered the expression of genes regulating pathogen detection in the peripheral blood molecular cells [56] and thus MOS may enhance disease resistance in pigs. 

Based on the discussed data it can be concluded that in the most critical period, right after weaning, dietary MOS supplementation may boost the immune response of the pigs and save nutrients for growth in case of infection.

## 5. The Effect of Dietary MOS on the Growth Performance of Weaned Pigs

Subject to the duration of starvation in the post-weaning period, the growth performance and the immune defense of the piglets suffer to a different extent. Some data show that the growth rate right after weaning has a significant impact on the pig performance in the growing and fattening phases [58]. Although the pig has the ability for compensation, the bigger the stress the less the chance is for recovery. This is certainly true for intensive genotypes. Therefore, any feeding strategy or feed supplement that alleviates the reduction in growth rate post weaning may have a positive effect on the efficiency of pork production.

The real growth promoter effect of MOS in nursery pigs is apparently inconsistent. Some studies report no benefits [25,26,59], while others found an improved rate of daily gain and/or feed efficiency in weaning pigs [24,48,60]. The results appear to be better in the case of younger pigs, especially in a challenged environment [8,61]. Based on the above-mentioned mode of action, the effect of MOS supplementation on piglet performance is affected by different factors, principally by weaning age, health status, duration of feeding and the amount of MOS addition. If the piglets are weaned at 4 weeks of age—as it is commonly done in the European Union, or even earlier, as in the United States—the intestine is less matured and the weaning is associated with a higher rate of gut epithelial atrophy than at a later age. Thus in the case of epithelial atrophy the positive effect of dietary mannan on gut wall repair can be demonstrated. Accordingly, the pig performance, e.g., growth rate and/or feed conversion were reported to be significantly enhanced in studies where the gut cell wall atrophy was reduced by dietary MOS supplementation [34,35,36]. 

In a meta-analysis involving studies with 54 comparisons, Miguel *et al.* [61] found that dietary MOS supplementation improves the growth rate mainly in the first 2 weeks of nursery, which is in agreement with the results showing that dietary mannans are associated with enhanced gut integrity in the post-weaning period. The same report suggests that pigs that received 1 or 2 g of the used mannan product (Bio-Mos) per kg of feed had a more pronounced growth response than pigs fed diets with supplementation of 3 or 4 g/kg [61]. This fact is supported by our findings as well; *i.e.*, in contrast to 4 g/kg, 1 or 2 g of a MOS product (AgriMos) per kg of feed increased the ileal digestibility of nutrients [41], as discussed earlier in this paper.

Recent studies conducted on any growth enhancer, in general, are in accordance with the earlier antibiotic growth promoter studies: under higher environmental pressure the treatment results in a better improvement of the zootechnical parameters (such as the average daily gain, feed conversion), however, the growth promoting effect is low if the animal performance is close to the genetic potential [62]. It has to be noted, however, that the activated immune system requires extra nutrient supply, therefore less amino acids and energy is available for growth in case of an immune challenge. Considering that dietary MOS supplementation has the potential to boost immune functions and defense mechanisms of the pig, the fact that the higher immune response is usually not associated with a lower growth rate is a benefit *per se*. 

## 6. Conclusions

Based on the relevant literature it is likely that mannans help to maintain the intestinal integrity and the digestive and absorptive function of the gut post-weaning. Therefore the malabsorption syndrome associated with this period can be alleviated with dietary MOS supplementation. Recent results suggest that MOS enhances the disease resistance in swine by promoting antigen presentation, thus enhancing the shift from an innate to an adaptive immune response. Accordingly, dietary MOS supplementation may have a growth promoting effect in pigs kept in a poor hygienic environment, while the positive effect of MOS is not observed in healthy pig herds with high hygienic standards, that are able to maintain their high growth rate post-weaning. In addition to the economic benefits resulting from the maintenance of gut health and support of the defense mechanisms, the use of medication and drugs can be reduced during the post-weaning period with the use of dietary MOS supplementation that enables healthier and safe, and therefore more desirable pork production.
